# The effect of minimum volume standards in hospitals (MIVOS) — protocol of a systematic review

**DOI:** 10.1186/s13643-022-02160-7

**Published:** 2023-01-20

**Authors:** Julia Scharfe, Stefanie Pfisterer-Heise, Charlotte Mareike Kugler, Eni Shehu, Tobias Wolf, Tim Mathes, Dawid Pieper

**Affiliations:** 1 Faculty of Health Sciences Brandenburg, Center for Health Services Research Brandenburg, Institute for Health Services and Health Systems Research, Brandenburg Medical School Theodor Fontane (MHB), Neuruppin, Germany; 2 Department of Medical Statistics, University Medical Center Georg-August-University Göttingen, Göttingen, Germany

**Keywords:** Minimum volume standard, Minimum volume regulation, Volume-outcome relationship, Centralization

## Abstract

**Background:**

The volume-outcome relationship, i.e., higher hospital volume results in better health outcomes, has been established for different surgical procedures as well as for certain nonsurgical medical interventions. Accordingly, many countries such as Germany, the USA, Canada, the UK, and Switzerland have established minimum volume standards. To date, there is a lack of systematically summarized evidence regarding the effects of such regulations.

**Methods:**

To be included in the review, studies must measure any effects connected to minimum volume standards. Outcomes of interest include the following: (1) patient-related outcomes, (2) process-related outcomes, and (3) health system-related outcomes. We will include (cluster) randomized controlled trials ([C]RCTs), non-randomized controlled trials (nRCTs), controlled before-after studies (CBAs), and interrupted time-series studies (ITSs). We will apply no restrictions regarding language, publication date, and publication status. We will search MEDLINE (via PubMed), Embase (via Embase), CENTRAL (via Cochrane Library), CINHAL (via EBSCO), EconLit (via EBSCO), PDQ evidence for informed health policymaking, health systems evidence, OpenGrey, and also trial registries for relevant studies. We will further search manually for additional studies by cross-checking the reference lists of all included primary studies as well as cross-checking the reference lists of relevant systematic reviews.

To evaluate the risk of bias, we will use the ROBINS-I and RoB 2 risk-of-bias tools for the corresponding study designs. For data synthesis and statistical analyses, we will follow the guidance published by the EPOC Cochrane group (Cochrane Effective Practice and Organisation of Care (EPOC), EPOC Resources for review authors, 2019).

**Discussion:**

This systematic review focuses on minimum volume standards and the outcomes used to measure their effects. It is designed to provide thorough and encompassing evidence-based information on this topic. Thus, it will inform decision-makers and policymakers with respect to the effects of minimum volume standards and inform further studies in regard to research gaps.

**Systematic review registration:**

PROSPERO CRD42022318883

**Supplementary Information:**

The online version contains supplementary material available at 10.1186/s13643-022-02160-7.

## Background

### Rationale

This protocol describes the rationale and methodology of a systematic review on minimum volume standards and the outcomes used to measure their effects. In the following, we present the background of the study, the existing literature on the topic, the methodology and study design, and the data synthesis. Finally, the discussion section outlines the implications for researchers as well as policy makers.

There is an ongoing debate in health care about whether a volume-outcome relationship exists. The term hospital volume-outcome relationship refers to the assumption that a certain health outcome (e.g., mortality or morbidity) is associated with hospital volume, i.e., higher hospital volume results in better health outcomes [1]. Hereby, hospital volume is defined by the number of cases a hospital has performed for a given procedure in a given time span. The presence of the volume-outcome relationship in surgery has been well established in the medical literature by our previous research [2, 3]. Yet, there are also many nonsurgical examples, such as dialysis, critical care, intensive care units, care for low-birth-weight infants, and care for people living with HIV/AIDS, where a volume-outcome relationship is likely to have been proven [4–8].

A possible explanation for this association is the “practice makes perfects” hypothesis, while other hypotheses are also discussed in the literature. According to the “practice makes perfects” hypothesis, more experience (i.e., higher volume of interventions) results in higher proficiency and better skills, and this should subsequently result in better patient-related outcomes. Nevertheless, the mechanism of why higher volumes result in better outcomes is not clearly understood.

Hospital volume has to be treated as a proxy measure [9], driven by other factors that can explain the hospital volume-outcome mechanism. Specific processes of care are regarded as the most probably explanatory factors. However, processes of care are multifactorial and therefore difficult to describe. For the example of surgical interventions, many processes are involved including preoperative and postoperative care. Structural characteristics might also have an influence on the outcome.

A recent scoping review investigated intermediate variables that might be able to explain the hospital volume-outcome relationship [10]. The authors categorized their results into three categories: compliance with evidence-based processes of care; level of specialization, such as specialized units and/or physicians (general surgeon versus specialized surgeon); and hospital level factors, such as available clinical services and nurse-to-patient ratio. All three categories have been confirmed as explanatory factors. However, no conclusion can be drawn as to which factors are the most important due to heterogeneous results.

If the “practice makes perfect” hypothesis holds true, health policy measures should include defining cutoffs for providers (i.e., minimum volume standards). A cutoff is a threshold in terms of a minimum number of procedures performed. This minimum volume standard is a necessary precondition for a provider to be allowed to perform this procedure in the future. Such measures have been implemented in a number of countries. The volume-outcome relationship can be described as an association in epidemiological terms. The minimum volume regulation is an intervention based on this relationship. Our systematic review focuses on examining the effect of this intervention.

### Minimum volume standards in Germany

A recent study found that besides Germany, minimum volume standards are in effect in Canada, the Netherlands, England, France, Italy, the USA, and Switzerland [11]. Those minimum volume standards differ in their design, yet they all define a certain threshold of procedures for a certain set of medical conditions which should be met in a given amount of time by the provider (hospital) of those medical procedures. In Germany, minimum volume standards were first implemented in 2004. Some of the minimum volume standards are implemented and increased stepwise (Table 1).

**Table 1:** Decided and pronounced minimum volume standards in Germany, 07/2022

Indication	Minimum volume (n)
Esophageal surgeries	*n* = 26 (*n* = 10 in 2022)
Pancreatic surgeries	*n* = 20 (*n* = 10 in 2022 and 2023)
Liver transplantation	*n* = 20
Kidney transplanation	*n* = 25
Stem cell transplantations	*n* = 25
Total knee replacements	*n* = 50
Care of low-birth-weight neonates	*n* = 25 (*n* = 14 in 2022, *n* = 20 in 2023)
Surgical treatment of breast cancer	*n* = 100 (no minimum volume in 2022 and 2023; *n* = 50 in 2024)
Thoracic surgical treatment of lung carcinoma in adults	*n* = 75 (no minimum volume in 2022 and 2023; *n* = 40 in 2024)

Source: Regulations of the Federal Joint Committee pursuant to Section 136b (1) Sentence 1 Number 2 SGB V for § Section 108 of the German Social Code, Book V, 16 July, 2022

Coronary artery surgery is also included in the list of procedures with a minimum volume standard, but no minimum volume standard has yet been defined. In the last years, the Federal Joint Committee (Gemeinsamer Bundesausschuss) commissioned the Institute for Quality and Efficiency in Health Care (IQWiG) to investigate the hospital volume-outcome relationship for several established procedures such as liver transplantations, stem cell transplantations, pancreatic surgeries, and esophageal surgeries but also for new procedures such as lung cancer surgery and breast cancer surgery. In addition, the rules of procedure (Verfahrensordnung) for minimum volume standards have been modified by the end of 2017. It has been made clear that there is no need to prove a causal effect for a hospital volume-outcome relationship. Instead, it is sufficient that the evidence suggests that such a relationship is likely. This is a serious change as the former rules of procedure relied heavily on the proof of a causal effect. However, this is hardly achievable when taking into account that randomized trials (with clustering) are very difficult, if not impossible, to conduct in this area. This has led in the past to a number of legal proceedings. For example, the minimum volume standard for total knee arthroplasty has been introduced in 2006, has then been interrupted for several years due to a legal dispute, and has only been reintroduced in 2015. Thus, it can be expected that the new rules of procedure will result in minimum volume standards for a greater number of procedures in the near future, as already announced by the German Ministry of Health [12].

However, when implementing minimum volume standards, it is important not to rely solely on the existence of a hospital volume-outcome relationship. In particular, from a patient’s perspective, factors such as regional availability and accessibility of inpatient services should also be taken into account.

An analysis of travel times revealed substantial regional differences for different procedures with minimum volume standards in Germany [13]. In May 2020, the Federal Joint Committee has commissioned the Institute for Quality Assurance and Transparence in Health Care (IQTiG) to investigate travel distance and travel time for patients for newly planned minimum volume standards (https://iqtig.org/veroeffentlichungen/folgenabschaetzungen-mm/).

### Recent evidence

A rapid review of the effects of minimum volume standards has been published by the IQWiG in 2012 [14]. In total, 10 studies dealing with the effects of minimum volume standards have been included. All included studies were observational, and the majority of them were before-after studies with only one time point of data collection before and one time point of data collection after the implementation of minimum volume standards. Nowadays, it is widely accepted that such studies should not be accepted to evaluate health policy measures as two time points do not allow for the calculation of time trends [15].

Besides this limitation of the evidence base in the IQWiG report, it must be borne in mind that the report was published as a rapid report. This means that it has inherent methodological shortcomings, while it was possible to publish the report within a shorter timeframe. Rapid review approaches typically involve updating the literature search of previous reviews, limiting the search strategy by date of publication, and having only one reviewer screen, extract data, and assess the quality of studies [16]. A recent meta-epidemiological study found that rapid reviews may produce different results to systematic reviews [17].

Nevertheless, the rapid report published by the IQWiG concluded that the included studies had very heterogeneous results with respect to patient-related outcomes such as mortality and morbidity. Interestingly, it found that hospital volume increased across hospitals, while the number of hospitals remained stable. This is a clear indication that hospitals might be willing to increase their case numbers in order to meet the minimum volume standards, even when there is no medical indication for this. This potential unintended effect has been confirmed in subsequent studies after the rapid report was published. The impact on travel times heavily depended on the procedure under study. In the end, the rapid report concluded that the study quality was too low, and results were too heterogeneous to draw any conclusions with respect to the effects of minimum volume standards on travel time.

When checking PubMed, we only found three relevant systematic reviews. One systematic review focuses on trauma care services [18]. Although 24 studies were included, only two of them had an appropriate methodological design. The second systematic review deals with prenatal care services [19]. Again, the majority of the included studies had no appropriate study design to allow drawing useful conclusions. The most recently published systematic review regarding minimum volume standards focuses on day surgery and includes 8 retrospective studies, which are also described as poor quality [20]. No other systematic evidence syntheses are available. An analysis of the impact of the whole legislation of setting minimum volume standards for all procedures in Germany has only been published in 2009 [9]. When comparing the procedures for which minimum volume standards are in place in Germany to our collated evidence [3], we found that there is limited evidence supporting a hospital volume-outcome relationship for some procedures, while there are many procedures with strong evidence but without a minimum volume standard [10].

We also searched for protocols and registered titles in the CDSR (Cochrane Database of SRs) and PROSPERO (International Prospective Register of SRs) in March 2022 but did not identify any ongoing systematic reviews on the topic.

Thus, we aim to investigate the effects of minimum volume standards in hospitals.

## Methods/design

### Objective

The objective of the systematic review is to gather encompassing information on the outcomes linked to the implementation of minimum volume standards in hospitals. Those outcomes can be related to the patient-level (e.g., mortality, morbidity, quality of life, functional measures, postoperative complications, travel times, distance to hospitals), the process-level (e.g., quality indicators), or the health system level in general (e.g., costs).

### Eligibility criteria

#### Population

We will include studies dealing with patients irrespective of their condition or the intervention received. All procedures performed within hospitals will be eligible for inclusion. Although it can be expected that the majority of procedures will be surgical, there will be no restriction to surgical procedures only. Other procedures such as care for acute myocardial infarction, pneumonia, or low-birth-weight neonates, for example, will also be included.

#### Intervention

The intervention is minimum volume standard, defined as a minimum of specific healthcare procedures in a given timeframe and area. In broad terms, we will include any change in how, when, and where healthcare is organized and delivered and who delivers healthcare if this involves the implementation of minimum volume standards. Minimum volume standards must be regarded within the context of a healthcare system. They can be expected to be different across states or regions. Thus, there will be no restrictions with respect to the regulatory approach (e.g., state authority, regional authority, and professional association), the year of implementation, selected standards or cutoff points, and consequences in case of noncompliance (e.g., non-reimbursement for the performed procedure).

#### Comparator

The comparator is no minimum volume standard.

#### Outcomes

We will consider a broad range of outcomes. The outcomes will be split into two groups (direct and indirect outcomes). For direct outcomes, we will largely follow the categorization scheme developed by the Cochrane EPOC group. As the included studies will cover a broad range of procedures, not all outcomes will be relevant for all procedures. In particular, patient outcomes will be amended depending on the procedure under study.

- Patient outcomes: (hospital) Mortality, survival, morbidity, health-related quality of life, functional measures, reoperations, wound infections, bleeding, postoperative complications, readmissions, length of stay
- Quality of care: Adherence to clinical practice guidelines or clinical pathways and quality assurance measures
- Utilization, coverage, or access: Travel times and distance to hospital
- Resource use: Costs
- Healthcare provider outcomes: Transition frequencies (i.e., number of hospitals increasing their volume to meet minimum volume standards) and number of procedures (e.g., due to broader indications)
- Adverse effects or harms: Any in one of the abovementioned categories

Any other outcomes not listed above will also be included.

Indirect outcomes will usually not be measured for a given procedure under study but at the health system level. As for any other health system intervention, the effects of minimum volume standards also need to be investigated at a structural level, as changes at the structural level can heavily affect any direct outcomes. These effects can be described as unintended. Nevertheless, this does not mean that they are less important. Some of these effects have already been described in the literature [21].

One such effect is procedure shifting (i.e., hospital focus on the delivery of new procedures). Hospitals not able to meet a minimum volume standard replace this procedure by focusing on the delivery of another procedure. Procedure shifting is acceptable unless broader indications are applied to the new procedure.

Minimum volume standards might also have an unintended effect on the delivery of emergency care. Hospitals not being able to deliver a procedure because of minimum volume standards might lack sufficient experience in case of emergency procedures. The German Medical Association has also pointed out that centralization might increase the shortage of young doctors in smaller hospitals due to an increased demand in high-volume hospitals [22].

Their report also concluded that overall, the effects at the health system level remain largely unstudied. There can be several such relevant effects at the same time, but no consensus on them has yet been reached.

All outcomes (direct and indirect) will be prioritized prior to conducting the review. Outcomes will be grouped into primary and secondary outcomes. This will be mentioned in amendments to the protocol.

#### Design of primary studies

We will include the following study designs:

- (Cluster) randomized controlled trials ((C)RCTs) with at least two intervention and control sites
- Non-randomized controlled trials (nRCTs) with at least two intervention and control sites
- Controlled before-after (CBA) studies with at least two intervention and control sites
- Interrupted time series (ITS) that have a clearly defined point in time when the intervention occurred and at least three data points before and three after the intervention.

RCTs (including cluster RCTs) are often not available to address questions about health system interventions and implementation strategies, such as minimum volume standards. As randomization in healthcare systems is very challenging, we do not expect any CRCT to meet our inclusion criteria. Therefore, we will include other study designs as suggested by the Cochrane Effective Practice and Organization of Care Group (EPOC). Following their guidelines, we will only include studies that have at least two sites (e.g., hospitals) included in each arm of the investigation. In a CBA, outcomes of interest are measured in both intervention and control groups before the intervention is introduced and after the intervention has been introduced. In an ITS, multiple data points are collected before and after the intervention, while the intervention effect should be measured against the pre-intervention trend. Therefore, ITSs should have at least three time points before and after the intervention. This study design does not require a control group. The study design will be determined using the Cochrane EPOC group algorithm.

Modelling studies (e.g., modelling the impact of centralizing procedures in a region to a smaller number of hospitals) will be excluded.

### Information sources and search strategies

We will conduct a literature search to identify all published and unpublished studies. The search strategy will be developed by the research team in collaboration with an experienced librarian and checked by a referee according to the Peer Review of Electronic Search Strategies (PRESS) guideline [23]. We will apply no restrictions regarding language, publication data, and publication status. A draft of the Embase search strategy is presented below:

((centralization:ti,ab OR centralisation:ti,ab OR centralized:ti,ab OR centralised:ti,ab OR regionalization:ti,ab OR regionalisation:ti,ab OR regionalized:ti,ab OR regionalised:ti,ab OR hospital volume*:ti,ab OR minimum volume*:ti,ab OR high case numbers:ti,ab OR minimum numbers:ti,ab OR minimum quantities:ti,ab OR minimal provider volume:ti,ab OR minimum provider volumes:ti,ab OR procedure volume*:ti,ab OR minimum hospital volume:ti,ab OR minimal hospital volume:ti,ab OR minimum volume standard*:ti,ab OR minimal amounts:ti,ab OR minimum amounts:ti,ab OR minimum patient volume:ti,ab OR hospital procedure volume:ti,ab OR hospital procedural volume:ti,ab OR high hospital volume:ti,ab OR minimum requirements:ti,ab OR minimum workload:ti,ab OR minimum caseload*:ti,ab OR case load requirement*:ti,ab) AND (hospital*:ti,ab OR clinic*:ti,ab OR center*:ti,ab OR centre*:ti,ab)) OR high volume hospital*:ti,ab OR ‘high volume hospital’/exp

*AND*

(law:ti,ab OR policy:ti,ab OR intervention*:ti,ab OR regulation*:ti,ab OR initiative*:ti,ab OR program*:ti,ab OR implementation*:ti,ab)

We will search the following databases to identify relevant studies: MEDLINE (via PubMed), Embase (via EMBASE), CENTRAL (via Cochrane Library), CINHAL (via EBSCO), EconLit (via EBSCO), PDQ evidence for informed health policymaking, Health Systems Evidence, and Open Grey. All databases will be searched without limitations to language, date, or land of origin. We will further search manually for additional studies by cross-checking the reference lists of all included primary studies as well as cross-checking the reference lists of relevant systematic reviews.

Furthermore, we will search the following trial registries: clinicaltrials.gov, German Clinical Study Register (DRKS), and International Clinical Trials Registry Platform (ICTRP).

We will also contact experts for additional studies and will conduct a hand search of available abstracts from conference reports.

### Data management and study selection

All potentially relevant hits will be imported to a reference management software (e.g., Rayyan). Duplicate publications will be removed. Two reviewers will independently screen titles and abstracts of all identified articles. We will retrieve the full texts of all potentially relevant articles. Full-text articles will be reviewed in detail regarding inclusion criteria by two reviewers independently. In case of disagreement, eligibility will be determined by discussion and consensus. In case of any uncertainty, we will contact the authors of the primary studies.

Based on preliminary searches, we expect to include 25 to 30 studies meeting our eligibility criteria.

A standardized data extraction tool will be developed in Excel and calibrated with the team. Using a random sample of five of the included studies, the data extraction form will be pilot-tested, and revised, as necessary. Data extraction will begin when high inter-rater reliability (Kappa statistic ≥ 0.60) has been achieved [24].

### Data collection and quality assessment

Two review authors will independently perform data extraction of the included studies using the standardized and piloted data collection form. Then, both reviewers will check each other’s versions for completeness and accuracy. Any discrepancies will be resolved by discussion. If no agreement can be reached, arbitration will be carried out by the senior researcher. Primary study authors will be contacted in case of missing data or uncertainty (e.g., follow-up time points). We will extract data on the following items: sample size (number of included patients and hospitals); study design; patients/hospitals eligibility criteria; type of hospitals (e.g. teaching hospital); surgeon characteristics (if applicable); year(s) of data collection; country/region; data source (clinical vs. administrative); database/registry (if any); procedure or treatment; definition of minimum volume standard; outcomes; (unadjusted and adjusted) effect measures with corresponding confidence intervals and/or *p*-values; statistical models; and adjusting variables.

For quality assessment, we will use the ROBINS-I risk-of-bias tool, with additions for CBA and ITS study designs. The ROBINS-I tool is a tool to assess non-randomized studies of interventions and includes seven domains. The first two domains cover confounding and selection of participants, addressing issues before the start of the interventions. The third domain addresses classification of the interventions themselves. The other four domains cover issues after the start of interventions: biases due to deviations from intended interventions, missing data, measurement of outcomes, and selection of the reported result. Judgements are “low risk,” “moderate risk,” “serious risk,” and “critical risk” of bias [25].

To assess the risk of bias of [C]RCT and nRCT studies, we will use the RoB 2 risk-of-bias tool. RoB 2 is results based and structured into a fixed set of domains of bias. Those domains include trial design, conduct, and reporting. The proposed judgement about the risk of bias arising from each domain is algorithm generated. Judgement can be “low” or “high” risk of bias or can express “Some concerns” [26].

Two reviewers will independently assess the risk of bias and resolve any disagreements through discussion. In case of insolvable disagreement, a third reviewer will be involved.

### Data synthesis

Our data synthesis strategy takes both methodological and clinical heterogeneity into account. From a methodological perspective, we will distinguish between different study designs and estimation approaches. From a clinical perspective, we will ensure medical homogeneity of studies considered for evidence synthesis.

For data synthesis and statistical analyses, we will follow the guidance published by the EPOC Cochrane group [27]: For dichotomous outcomes, we will use the risk ratio (RR) obtained from statistical analyses adjusting for baseline differences (such as Poisson regressions or logistic regressions) or the ratio of risk ratios (i.e., the RR post-intervention/RR pre-intervention), if possible. For continuous outcomes, we will use the absolute change obtained from a statistical analysis that has adjusted for baseline differences (e.g., regression models, mixed models, or hierarchical models). Alternatively, we will use the relative change adjusted for baseline differences in the outcome measures. This is the absolute post-intervention difference between the intervention and control groups minus the absolute pre-intervention difference between the intervention and control groups. For ITS studies, if possible, we will rely on the results either obtained by a regression including time trends before and after the intervention adjusting for autocorrelation and any periodic changes or auto-regressive integrated moving average (ARIMA) models. Results of interest refer to both, change in slope and change in level. Change in slope is the change in the trend from pre- to post intervention, while change in level refers to the immediate effect of the intervention. The immediate effect is calculated by the difference between the fitted value for the first post intervention data point minus the predicted value based on the pre-intervention slope only.

If papers with ITS design do not provide an appropriate analysis or reporting of results, but present the data points in a readable graph or in a table, we will reanalyze the data using a segmented time series regression model: Y(t) = B0 + B1*preslope T + B2*postslope (T – Ti)+ B3*intervention Xt + e(t) as suggested for Cochrane EPOC reviews, where Y(t) is the outcome in month t. Pre slope is a continuous variable indicating time from the start of the study up to the last point in the pre-intervention phase and coded constant thereafter. Post slope is coded 0 up to and including the first point post intervention and coded sequentially from 1 thereafter. Intervention is coded 0 for pre-intervention time points and 1 for post intervention time points. In this model, B1 estimates the slope of the pre-intervention data, B2 estimates the slope of the post intervention data, and B3 estimates the change in level of outcome as the difference between the estimated first point post intervention and the extrapolated first point post intervention if the pre-intervention line was continued into the post-intervention phase. The difference in slope is calculated by B2-B1. The error term e(t) is assumed to be first-order autoregressive. Confidence intervals (95%) will be calculated for all effect measures.

Analysis will be performed at the same level as the allocation to avoid unit-of-analyses errors. If such results will be reported in the included studies, and there is insufficient data to reanalyze the data, we will try to obtain data by contacting study authors. If these data will not be available, we will not report CIs and *p*-values for analyses containing unit-of-analyses errors.

If there are sufficient numbers of comparisons for similar outcomes and similar procedures across studies, we will use “box and whisker” plots to graphically display and explore heterogeneity of the results across studies. In addition, we will use *I*^2^ statistic to assess the extent of variability beyond chance for each of the groups of studies assessing similar comparisons, outcomes, and procedures.

For data synthesis, we will prepare a table for each category of interventions and procedures. Categories for interventions are hardly to be determined before. Therefore, we will judge on defining categories in view of the identified studies. Categories for procedures will consist of single procedures (e.g., total knee arthroplasty). However, if there is enough evidence from other studies suggesting the same effects across different procedures in a given field, we might decide to merge them into one category (e.g., all procedures in knee arthroplasty).

For all studies, we will record the number of events (in the case of health outcomes) and the total number in each group (for risk ratios) or mean and standard deviation (SD) in each group (for mean difference). All outcome effects will be shown with their associated 95% CIs.

We will only conduct a meta-analysis for studies that report similar comparisons (procedures, interventions, comparisons, and outcomes that are similar enough that an average effect across those studies would be meaningful).

Anticipating heterogeneity across studies, we will use a random-effects model for meta-analysis.

For CBAs and ITS, we will pool changes in intercept and slope.

In the case that no meta-analysis will be performed, a structured synthesis as suggested by EPOC will be conducted. We will describe the range of effects in the identified studies. Furthermore, we will describe the underlying mechanism through which the intervention affects specific outcomes, if feasible.

Subgroup analyses will be performed for different interventions, health systems (i.e., countries, regional health authorities), and procedures or corresponding categories.

We will perform sensitivity analyses for missing data by imputing a plausible range of assumptions. The potential implications of missing data will be discussed. We will also perform sensitivity analyses for different study types and differing risks of bias. Studies at a high risk of bias will be excluded from sensitivity analyses.

For data synthesis, we will use R in its current version.

### Certainty assessment

Quality of the evidence will be evaluated using the Grades of Recommendation, Assessment, Development and Evaluation (GRADE) approach [28]. The GRADE approach uses five considerations (study limitations, consistency of effect, imprecision, indirectness, and publication bias) to assess the quality of the body of evidence for specific outcomes. Although GRADE has originally been developed for clinical questions, it can also be applied to public health or health system questions, albeit it is likely that this might be challenging. Currently, the GRADE working group is underway to identify challenges in applying the GRADE approach to public health and health system interventions to come up with potential solutions at a later stage. Evidence can be downgraded depending on assessments for risk of bias, indirectness of evidence, serious inconsistency, imprecision of effect estimates, or potential publication bias. The quality of the body of evidence will be assessed by two reviewers independently using the GRADEpro GDT software. Summary of finding tables will be prepared for summarizing confidence across studies.

## Discussion

This systematic review will provide an encompassing and thorough overview of the variety of effects of minimum volume standards in hospitals. Up to date, systematic analyses of the effects of minimum volume standards in hospitals are scarce and struggle with the poor quality of the included primary studies as well as with the narrow focus on patient-related outcomes. We defined strict inclusion criteria regarding the study types that can be included in this review in hope of thereby improving the overall quality of the evidence of this systematic review. This systematic review aims at broadening the perspective and providing an encompassing overview of the variety of effects deriving from minimum volume standards in hospitals. Thus, it will inform decision-makers and policy makers with respect to minimum volume standards and provide a source of knowledge for further studies regarding the topic of minimum volume standards.

It is important for systematic reviews to make their data available in the long term. By promoting open science and data sharing, efficiency in conducting systematic reviews can be expected to increase greatly. At the same time, unnecessary duplication resulting from recurring data extraction of the same primary studies included in multiple systematic reviews can be reduced. Therefore, we will use the Open Science Framework (OSF) to organize and store all data of our systematic review. Hereby, our data will be freely available to other researchers working on similar topic in the long term.

We aim to publish the results in an international journal with open access and in a German language journal to assure dissemination in Germany. We will provide all material as supplemental online material. The results will be also presented at international and national conferences to enhance the dissemination of results within important scientific communities (e.g., ISEHC (International Society for Evidence-Based Health Care), DNEbM (German Network Evidence-Based Medicine), or DNVF (German Network for Health Services Research). Furthermore, procedure-specific results will be disseminated to the corresponding German clinical societies (e.g., German Society of Surgery (DGCh)). In particular, we will also disseminate our results to countries/regions with minimum volume standards in place which have been recently identified [11].

## Supplementary Information

**Additional file 1.** PRISMA-P 2015 Checklist.

## Data Availability

The datasets generated and/or analyzed during the current study will be available in the Open Science Framework repository, https://osf.io.

## Abbreviations

[C]RCTs: (Cluster) randomised trials
nRCTs: Non-randomised controlled trials
CBAs: Controlled before-after studies
ITS: Interrupted time series
RCTs: Randomised controlled trials
EPOC Cochrane Group: Cochrane Effective Practice and Organization of Care Group
IQWiG: Institute for Quality and Efficiency in Health Care
IQTIG: Institute for Quality Assurance and Transparence in Health Care
SRs: Systematic reviews
CDSR: Cochrane database of SRs
PROSPERO: International Prospective Register of SRs
PRESS: Peer Review of Electronic Search Strategies
MEDLINE: Medical Literature Analysis and Retrieval System Online
Embase: Excerpta Medica Database
CINHAL: Cumulative Index to Nursing and Allied Health Literature
CENTRAL: Cochrane database
EconLit: Index of the American Economic Association
PDQ evidence for informed health policymaking: “pretty darn quick” evidence for informed health policymaking database
DRKS: German Clinical Study Register
ICTRP: International Clinical Trials Registry Platform
GRADE: Grades of Recommendation, Assessment, Development and Evaluation
RR: Risk ratio
ARIMA model: Auto-regressive integrated moving average model
OSF: Open Science Framework
ISEHC: International Society for Evidence-Based Health Care
DNEbM: German Network Evidence-Based Medicine
DNVF: German Network for Health Services Research
DGCh: German Society of Surgery
